# ﻿*Ficusmotuoensis* (Moraceae), a new species from southwest China

**DOI:** 10.3897/phytokeys.206.89338

**Published:** 2022-09-06

**Authors:** Zhen Zhang, Mei-Jiao Zhang, Jian-Hang Zhang, De-Shun Zhang, Hong-Qing Li

**Affiliations:** 1 College of Architecture and Urban Planning, Tongji University, Shanghai 200092, China Tongji University Shanghai China; 2 Qingpu Lansheng School, 1588 Zhujiajiao Road, Shanghai, 201713, China Qingpu Lansheng School Shanghai China; 3 School of Life Sciences, East China Normal University, Shanghai 200241, China East China Normal University Shanghai China

**Keywords:** climbing figs, fig tree, new taxon, Rosales, Sino-Himalaya

## Abstract

A new climbing species, *Ficusmotuoensis* Zhen Zhang & Hong Qing Li in Moraceae from southwest China has been described and illustrated in this paper. The new species resembles *F.disticha*, *F.diversiformis* and *F.hederacea*, but differs from these in the medium-sized acrophylls, shorter peduncle, as well as larger and spotted syconium. According to the morphological traits and phylogenetic placement, the new species belongs to Ficussubg.Synoeciasect.Apiosycea. Besides, the new species deviates from the common distribution pattern compared to the other members of sect. Apiosycea, indicating that it could be very useful for exploring the biogeography of sect. Apiosycea.

## ﻿Introduction

*Ficus* L. is an extremely species-rich woody genus in the family Moraceae, mainly distributed in tropical and subtropical regions (Berg and Corner 2005; Pederneiras et al. 2018; Zhang et al. 2020). As the largest genus in Moraceae, *Ficus* features the syconium and mutualism relationship to fig wasps (Janzen 1979; Wiebes 1979; Bronstein and McKey 1989). To date, the number of species in *Ficus* has grown to almost 800 after recent frequent descriptions from South America, Southeast Asia and so on (Chantarasuwan and Thong-Aree 2006; Berg and Homeier 2010; Whitfeld and Weiblen 2010; Berg 2012; Medina 2014; Machado and de Queiroz 2017; Pederneiras et al. 2017; Ezedin and Weiblen 2019; Rivera et al. 2020). Southwest China, especially the Himalaya-Hengduan Mountains, is one of twenty-five biodiversity hotspots worldwide and possesses abundant endemic species (Myers et al. 2000). Meantime, many gynodioecious fig trees are endemically distributed in the Himalaya-Hengduan Mountains or the Sino-Himalaya Region in Ficussubg.Ficus and subg. Synoecia (Miq.) Miq. However, relatively few species in *Ficus* have been described from these regions in two decades (Chen et al. 2020), indicating a possible underestimate of biodiversity.

Through two field investigations with an interval of seven years in Motuo County, Tibet, in China, we found an unrecorded climbing fig tree. The climbing species is rather distinct in the aspects of small leaves and spotted syconia compared to the other Chinese climbing figs. Based on specimen examination and phylogenetic analyses, we confirmed that it is a new species in subg. Synoecia and provided its taxonomical description and illustration.

## ﻿Materials and methods

### ﻿Morphological observations

The novel species was surveyed in Motuo County, Tibet, in China. The morphological characteristics were measured and then photographed by digital camera (Canon, D80) or stereomicroscope (SMZ25, Nikon). The type specimens have been stored in the Herbarium of the East China Normal University (**HSNU**). The morphological comparison between the new species and its congeners has also been examined.

### ﻿Phylogenetic inference

Three samples represented the new species and three nuclear loci, internal transcribed spacer (ITS), external transcribed spacer (ETS) and glyceraldehyde 3-phosphate dehydrogenase (*G3pdh*) were used to verify its phylogenetic placement in *Ficus*. Simultaneously, two samples of its morphologically related species *F.hederacea* Roxb. were supplemented. The other taxa and sequences in subg. Synoecia were selected according to the work of Zhang et al. (2020) and their GenBank accession numbers can be found in Suppl. material 1: Table S1. In total, twenty-eight samples were involved in phylogenetic analyses together with two extra samples of *F.laevis* Blume as the outgroups.

Bayesian Inference (BI) and Maximum Likelihood (ML) analysis were implemented to reconstruct the phylogenetic trees. Bayesian Inference was carried out by MrBayes 3.2.6 (Ronquist et al. 2012) with 5,000,000 generations, sampling every 1,000 generations to ensure the convergence (average deviation of split frequencies less than 0.01 and the effective sample sizes over 200). The first 25% of sampling trees were treated as burn-in and the remainder were used to prepare the consensus tree and posterior probabilities. The IQ-TREE 2.1.3 (Nguyen et al. 2015) was used to reconstruct the Maximum Likelihood tree with 10,000 ultrafast bootstrap to assess the confidence of the nodes. The nucleotide substitution models for both BI and ML were selected by ModelFinder (Kalyaanamoorthy et al. 2017) with the respective commands. The ML tree was chosen to show the topology after visualisation by Figtree 1.4 (http://tree.bio.ed.ac.uk/software/figtree/).

## ﻿Results

### ﻿Morphological observations

The novel species is a gynodioecious root-climbing taxon with obvious dimorphic leaves, thus it should belong to subg. Synoecia. Some key traits, including leaf dimorphy, rather small leaves, spotted syconia and sessile flowers, make the new species fairly distinct in subg. Synoecia. Four climbing fig trees, including *F.laevis*, F.pubigeravar.pubigera, F.pubigeravar.maliformis and *F.sarmentosa* Buch.-Ham. ex Sm., were sympatric with the new species in Motuo County, based on our field investigation. However, none of them resembles the new species. The bathyphylls of the new species are similar to those of *F.disticha* Blume (subg. Synoecia), except for the symmetric lamina and its round apex. Besides, the acrophylls (4.5–6.5 cm in length) and syconia (8–10 mm in diameter) of the new species are obviously larger than those of *F.disticha*. The new species also resembles *F.hederacea* and *F.diversiformis* in the aspect of acrophylls, whereas its syconia are different from these. The syconia of *F.hederacea* is globose with a 10–12 mm peduncle in length and those of *F.diversiformis* is basal constricted with a 3–12 mm peduncle. Both of them are clearly longer than the new species (1–2 mm). From a geographical point of view, the new species is also allopatric to *F.disticha*, *F.hederacea* and *F.diversiformis*. A comparison between the new species and its morphological allies are shown in Table 1.

**Table 1.** Morphological comparison amongst the new species and its three allies.

	*F.motuoensis*	*F.disticha*	*F.hederacea*	*F.diversiformis*
**Bathyphylls size (cm)**	1.5–2.5 × 0.8–1.2	1–2.5 × 0.8–1.5	4–8 × 2–3.5	1–2 × 0.5–1.5
**Acrophylls size (cm)**	4.5–6.5 × 2.5–3.5	2.5–5 × 2–5	6–11 × 3.5–5	1.5–5.5 × 1–3
**Acrophylls shape**	elliptical	variable, somewhat obovate	elliptical	obovate
**Veins (pairs)**	5–6	3–7	3–5	2–3
**Peduncle length (mm)**	1–2	0–4	10–12	3–12
**Syconia size (mm)**	8–10	3–6	7–14	10–13
**Syconia shape**	globose	globose to pyriform	globose	globose to pyriform
**Syconia colour**	green to red with variegation	green to red-brown to purplish	green to orange	unknown

### ﻿Phylogenetic Inference

**Figure 1.**
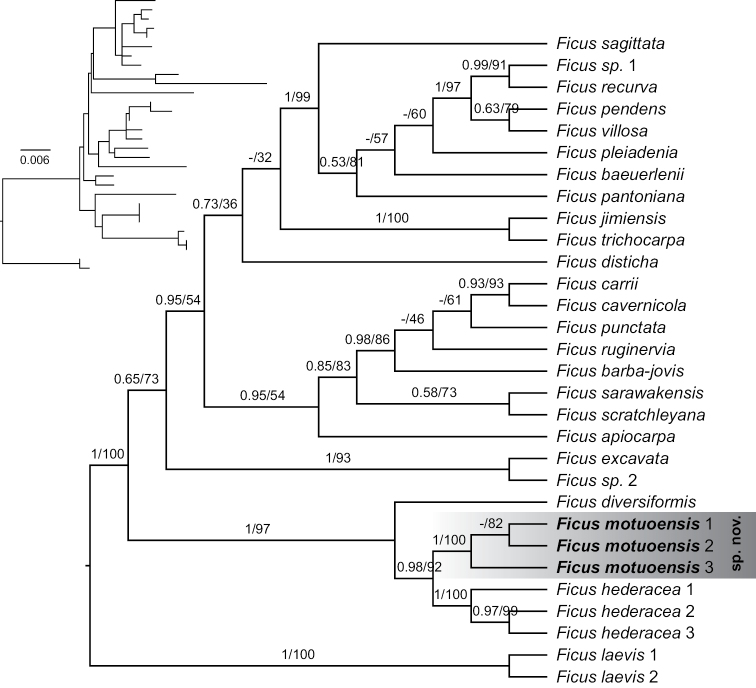
The Maximum Likelihood cladogram, based on three nuclear loci (ITS + ETS + *G3pdh*) with posterior probabilities and ultrafast bootstrap values shown on the branches. The phylogram of the tree shown in the upper left.

The phylogenetic tree indicates that three samples of the new species comprised a well-supported monophyletic group (posterior probability = 1 and ultrafast bootstrap value = 100, Fig. 1). The new species is phylogenetically sister to another widely distributed species *F.hederacea* and both of them form a sister relationship to the Sri Lankan endemic species *F.diversiformis*. The closely-related genetic relationship amongst these three species is also supported by their morphological similarity. Another morphologically related species *F.disticha* is far from the new species in the phylogenetic tree.

### ﻿Taxonomic treatment

#### ﻿ Ficus motuoensis

Taxon classificationPlantaeRosalesMoraceae

Zhen Zhang & Hong Qing Li sp. nov.

0CF26215-93C4-5BDA-86AE-45DE3C908ADD

urn:lsid:ipni.org:names:77304424-1

Figs 2 , 3

##### Type.

China. Tibet (Xizang): Linzhi, Motuo, Deergong. 25 Jun 2021, *Zhen Zhang & Jian-Hang Zhang ZZ966* (holotype HSNU00079864!, isotype HSNU00079862!); paratype *Zhen Zhang & Jian-Hang Zhang ZZ905* (HSNU00079863!), *Zhen Zhang & Jian-Hang Zhang ZZ955* (HSNU00079861!), *Zhen Zhang & Jian-Hang Zhang ZZ962* (HSNU00079865!, HSNU00079866!, HSNU00079867!).

##### Diagnosis.

*Ficusmotuoensis* is similar to *F.disticha* in the shape and texture of the bathyphylls, but differs from the latter by its larger acrophylls (4.5–6.5 cm in *F.motuoensis* versus 2.5–5 cm in *F.disticha*) and larger syconia (8–10 mm in *F.motuoensis* versus 3–6 mm in *F.disticha*). The new species also resembles *F.hederacea* and *F.diversiformis* in the aspect of the acrophylls, but can be distinguished from these by its globose and spotted syconia (versus without spots in *F.hederacea* and *F.diversiformis*) with a shorter peduncle (1–2 mm in *F.motuoensis* versus 10–12 mm in *F.hederacea* and 3–12 mm in *F.diversiformis*).

##### Description.

Gynodioecious root-climber. Branchlets densely pale pubescent, glabrous in biennial branches, with some lenticels in biennial branches. Stipules 2, 2–3 mm in length, long triangular-lanceolate, glabrous, reddish-brown, caducous; bathyphylls distichous, petiole 2.5–4 mm, greenish to light brown, adaxially furrowed, densely white pubescent at the both sides of furrow, lamina elliptical, 1.5–2.5 × 0.8–1.2 cm, symmetric, thinly chartaceous, base rounded, apex acute, margin entire, veins 4–5 pairs, abaxially slightly raised, basal vein up to 1/3 the length of the lamina, both surfaces glabrous, the abaxially surface tessellate; acrophylls distichous, petiole 5–10 mm, brown, subgrabrous, adaxially furrowed, lamina elliptical, 4.5–6.5 × 2.5–3.5(–4) cm, coriaceous, base rounded, apex acute to obtuse, margin entire, veins 5–6(–7) pairs, abaxially slightly raised, basal vein up to 1/3 the length of the lamina, both surfaces glabrous, the abaxially surface tessellate. Figs axillary on the leafy or leafless branchlets, in pairs or sometimes solitary; peduncle 1–2 mm, basal bracts 3, ca. 1 mm in length, broadly ovate, glabrous; receptacle globose, 8–10 mm in diameter when fresh, greenish to red when mature, densely covered by light-green speckles, glabrous; ostiole ca. 2 mm in diameter; internal hairs absent. Staminate flowers numerous, scattered, sessile; calyces 3–4, light pink, translucent, glabrous, ovate-lanceolate, ca. 2 mm in length; stamens 2–3, slightly shorter than calyx; anther oblong, ca. 1.2 mm in length, not mucronate; filament free, ca. 0.6 mm in length, a few hairs born on the joint of filaments. Gall flowers numerous, sessile; calyx 3–5, light pink, translucent, lanceolate to linear, 2–2.5 mm in length, glabrous; ovary elliptical, base constricted to being gynophore, 0.5–2 mm; style subapical, short, ca. 0.2 mm in length; stigma funnel-form, margin lacerate. Pistillate flowers not seen.

**Figure 2.**
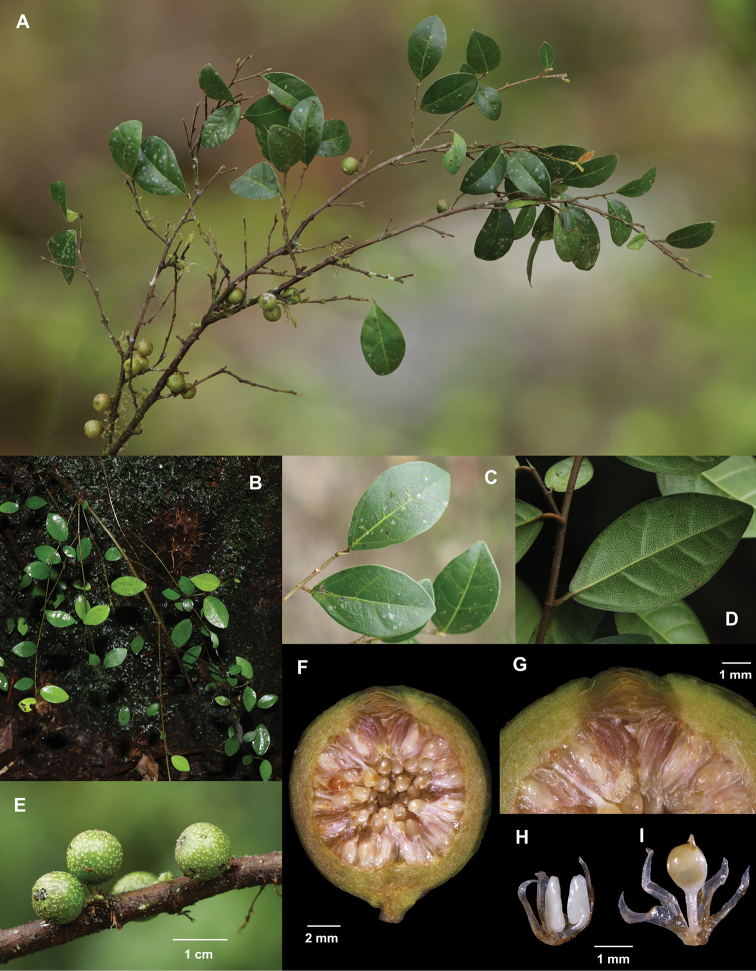
Illustration of *Ficusmotuoensis***A** fruit branch **B** vegetative branch **C** adaxial surface of acrophylls **D** abaxial surface of acrophylls **E** syconia **F** profile of staminate inflorescence **G** ostiole bracts **H** staminate flower **I** gall flower.

##### Chinese name.

Mo Tuo Rong (墨脱榕).

##### Etymology.

The specific epithet indicates its type locality, Motuo County, Tibet, in China.

##### Distribution and habitat.

Only found in the type locality, i.e. China: Tibet, Linzhi, Motuo County. However, considering that Motuo is close to Assam in India, the new species probably also exists in India. The new species develops very well in the type locality, as it has been recorded in five different villages (Bari, Yarang, Gelin, Deergong and Maniweng). The individuals of the new species are rather abundant without the risk of extinction. The new species grows in the tropical monsoon forest climbing on substrates, such as soils and tree trunks, whereas its fertile branches often break away from the substrate at the time of reproduction. It is located at an altitude of 700–2000 m.

##### Note.

Based on the morphological traits and phylogenetic placement, the new species is related to *F.disticha*, *F.diversiformis* and *F.hederacea*. In the latest division framework of *Ficus*, these three species were assigned to sect. Apiosycea (Miq.) Pedern. & Romaniuc (Zhang et al. 2020). However, the taxa in sect. Apiosycea are mainly distributed in Malesia (Zhang et al. 2020). In total, six Chinese taxa belong to sect. Apiosycea, including *F.hederacea*, *F.laevis*, *F.punctata* Thunb., *F.sagittata* Vahl, *F.trichocarpa* Blume and *F.villosa* Blume, but south China is only the northern limit of their distribution range (Chang et al. 1998; Berg and Corner 2005). Therefore, the new species, which is endemic to southwest China, could be very useful for exploring the biogeography of sect. Apiosycea.

**Figure 3.**
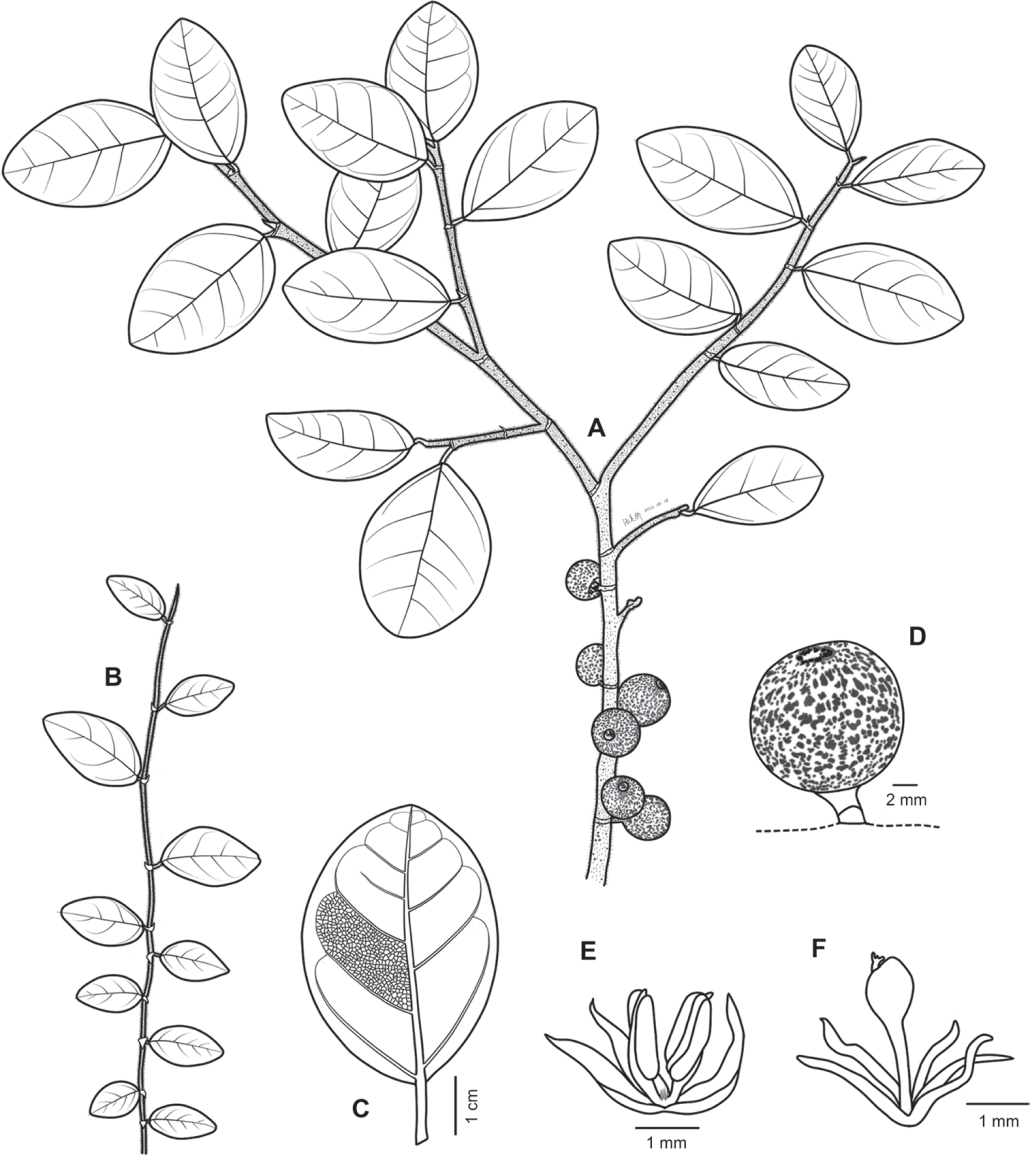
Illustration of *Ficusmotuoensis***A** fruit branch **B** vegetative branch **C** abaxial surface of acrophylls **D** syconium **E** staminate flower **F** gall flower.

## Supplementary Material

XML Treatment for Ficus motuoensis

## Supplementary materials

Supplementary material 1 Table S1 Data type excel file Explanation note Detailed information of samples in the study. https://binary.pensoft.net/file/739003This dataset is made available under the Open Database License (http://opendatacommons.org/licenses/odbl/1.0/). The Open Database License (ODbL) is a license agreement intended to allow users to freely share, modify, and use this Dataset while maintaining this same freedom for others, provided that the original source and author(s) are credited.Zhen Zhang, Mei-Jiao Zhang, Jian-Hang Zhang, De-Shun Zhang, Hong-Qing Li
